# Development of a nano-size off-grid energy system using renewables and IoT technologies at the Meteoria visitor center: A Finnish case study

**DOI:** 10.1016/j.heliyon.2023.e21473

**Published:** 2023-10-31

**Authors:** Jessica Tuuf, Hans Lindén, Sami Lieskoski, Margareta Björklund-Sänkiaho

**Affiliations:** a Åbo Akademi University, Faculty of Science and Engineering, Energy Technology, Rantakatu 2, 65100, Vaasa, Finland; b Novia University of Applied Sciences, Technobothnia, Puuvillakuja 3, 65200, Vaasa, Finland

**Keywords:** Off-grid, Energy system, IoT technologies, Raspberry PI

## Abstract

The increased utilization of non-renewable energy during the last century has influenced the climate, with increased carbon dioxide emissions and elevated temperature as a result. Thus, the need to develop and demonstrate new sustainable solutions regarding both energy supply and consumption, but also energy system optimization, is obvious. This case study presents the nano-size off-grid energy system at the Meteoria visitor center in Ostrobothnia, Finland, and the real-time measuring techniques that have been installed to follow up the energy production and consumption. The Meteoria consists of several buildings, which are open to the public from April to October. The case site is operated by energy derived from wind power, solar power, and a diesel generator (as a backup), with batteries for energy storage. The Internet of Things (IoT) has been retrofitted to the existing energy system to enable energy measurements and follow various electrical parameters in real-time. In addition, a graphical visualization platform open to the public has been developed. In this study, the completeness of data sampling and the IoT system was checked, and the results show high availability of data. Furthermore, various errors/limitations regarding the IoT system were identified. The energy supply/demand at the Meteoria in 2021 was monitored and the challenges regarding the existing energy system in a cold climate zone are discussed as well as the potential role of the Meteoria to function as a living lab.

## 1 Introduction

The utilization and availability of and dependency on various energy sources are topics that are of utmost importance in today's society. Furthermore, the ongoing environmental challenges and problems regarding elevated greenhouse gas emissions due to fossil fuel utilization, are forcing politicians, researchers, and citizens to re-evaluate the existing processes. To be able to cope with the elevated carbon dioxide emissions and to create a more sustainable society, increased usage of renewable energy sources (RES) is required. These sources include hydro-, solar- and wind power, biomass, ambient energy sources via heat pumps, but also energy stored in various gases, such as hydrogen. In 2020, worldwide electricity production was generated by renewables to an extent of 7,468 TWh. Of this, hydropower accounted for 59 %, wind power 21 %, solar power 11 %, bioenergy 8 %, and geothermal energy 1 % [1]. In Finland in 2021, 20.4 % of the electricity consumed was imported and 79.6 % of the electricity was produced domestically. Of this internal electricity, the four largest energy sources were nuclear energy (26.0 %), hydropower (17.9 %), wood fuels (13.9 %), and wind power (9.3 %). Solar power constituted 0.3 % and other sources 12.2 % [2]. Hydrogen, as a long-duration energy storage technology, an alternative carbon-free fuel in the transport and industrial sector, and an energy carrier has gained a lot of interest in the last years, and it is suggested that green hydrogen will play an imperative role in the future in achieving a net-zero emission energy system [3]. The increasing amount of installed wind power capacity in Finland will probably boost hydrogen production via electrolysis. By promoting sustainable energy solutions, several benefits can be obtained, including lowering the carbon footprint in various sectors and promoting issues such as energy security. Finland's national energy and climate strategy [4], EU-level policy initiatives like the European Green Deal [5], and the related legislative package called Fit for 55 [6] strongly support a rapid transition towards carbon-neutral energy systems. For example, within the building sector, the goal is that all new buildings should have zero emissions by 2030 and that existing buildings should be renovated and transformed into zero-emission buildings by 2050 [6]. Energy security issues and energy self-sufficiency are other highly prioritized focus areas in Europe and Finland. In 2022, the energy trade suddenly changed in Europe and there was a need to secure enough energy for the people, to promote new sustainable energy solutions, and to induce a change in energy consumption behavior. The REpowerEU plan, launched by the European Commission in 2022, also supports these actions [7].

### 1.1 Literature review

When introducing RES into an existing energy system, there are several issues to consider such as the energy source availability and intermittency, optimal sizing of components, energy storage possibilities, and the cost of investments. To examine these phenomena, off-grid systems can be utilized with advantage. An off-grid energy system could be defined as a system where there is no connection to the main electrical grid and where the self-sufficiency rate of energy of the system is remarkably high. The reasons for developing and maintaining off-grid energy systems might differ, however, limited availability to the electrical grid due to remote location, the drive to be self-sustaining in energy supply, energy security, and affordability are arguments often mentioned. One way of dividing off-grid systems is based on size, where a mini-grid operates with less than a 10 MW capacity, a micro-grid operates with less than 100 kW of capacity and the nano-grids usually consist of individual houses or buildings in isolated areas [8]. In remote areas, especially islands, the diesel generator has had a key role as a supplier of power. However, since the fuel transported to the site is expensive, and the utilization of fossil fuels contributes to carbon dioxide emissions, locally produced renewable energy has gained more interest. Due to the intermittent nature of wind and sun, there is generally a requirement for a combination of various RES and storage solutions, e.g., batteries, to enable a reliable power system. A cold winter climate will cause additional challenges for battery storage, as it must be heated to not lose storage capacity. Numerous technical and/or techno-economic studies have been conducted on different combinations of hybrid systems, e.g., PV (photovoltaics)/wind/batteries [9], wind/diesel/batteries [10,11], or PV/wind/diesel/batteries [12]. The goal has been to find an optimized solution for the utilized energy sources in a specific case, both based on cost and technical conditions. For example, in the Philippine archipelago, a study was performed on remote islands, looking at the specific features each island possessed and classifying the energy system based on those features. The results showed that the PV/battery system had the most potential for a future energy system in that area [13]. In a case study from Iran, various hybrid systems were modeled for analysis of optimal performance and cost-effectiveness, and the result showed the wind/diesel/battery combination to be the most cost-effective in this specific area [14]. In addition to batteries, other forms of energy storage in off-grid systems have been analyzed, e.g., hydrogen storage. A hybrid system with PV and hydrogen storage in a residential house in Mexico has been modeled and integrated with success [15]. The selection of the right parameters for analysis is crucial. For example, a feasibility study was performed in Malaysia to explore integrating solar power in an off-grid area using the HOMER system modeling software as the investigating tool. Several important parameters were identified, such as total energy production, average energy consumption, and greenhouse gas emissions. Also, the climatic conditions at the site, in this case, e.g., the solar radiation availability, were suggested as crucial [16]. The daily and seasonal characteristics of the energy supply and the load profile are preferable knowledge when optimizing an off-grid hybrid power system, which is also shown in a case study of west China [17]. These parameters helped in deciding an optimal energy system solution that was cost-effective in that specific environment. Altogether, these are just a few examples of the potential various hybrid solutions can offer, both regarding economic parameters as well as technical ones.

In practice, the environmental conditions and the specific geographical location are factors that must be considered when planning an energy system outside the main grid. The climate, the seasonal variations, and the availability of renewable sources at the site are all crucial for maintaining a sustainable power system. Locations in northern climates will put restraints on the energy system because the temperature can vary substantially for one day but also throughout the year. Annual temperature variations from −35 °C to +35 °C are not rare. The functioning of solar PVs, solar thermal (T), and PV/T in cold climates has previously been discussed [18]. Here, several parameters were suggested to play a vital role in the various solar energy technologies, including sensitivity to temperature, dust interference, and solar tracking performance. It is well established that PV panels are affected by temperature since the electrical efficiency of PV panels and the power output of the PV panels depend on the operating temperature [19]. A study conducted in Serbia has compared different theoretical models of assessing PV performance with experimentally measured values [20]. Here, it was shown that the PV panels had the highest efficiency in December, which is considered the coldest month, compared to the summer months of July and August. In a performance evaluation study on PV panels in an off-grid system in Saudi Arabia, Rehman et al. [21] showed that PV panels had a decreased performance when the temperature of the PV panel surface was increased during the hottest hours during the days. However, the decreased efficiency of PV panels with increasing operating temperature can be counteracted with various methodologies, such as water or air cooling, heat pipe cooling, and cooling with phase change materials [22]. In northern climates, the possible gain in efficiency during the cold winter months might not fully be taken advantage of due to poor solar radiation. Another challenge with PV panels in cold-climate regions is snow coverage. By looking at PV energy systems at six distinct locations in Wisconsin and Colorado, the impact of snow coverage on PV panels (PV tilt ranging from 15° to 35°) performance was analyzed [23]. The results indicated that the monthly average energy losses due to snow coverage were as high as 90 %. Another study in Canada has developed a predictive model, based on an artificial neural network, predicting the daily energy production of numerous PV systems in cold-climate regions in the northern hemisphere [24]. It was proposed that it is more advantageous to have PV panels with higher tilt angles (50°or 60°) than low tilt angles because the snow will then more easily slide off and because of the northerly latitude. Data-based machine learning models can be applied to optimize renewable energy systems, for instance, to predict the remaining useful life of lithium-ion batteries [25]. Gopi and coworkers (2022) applied three different data-based AI techniques, using weather-related input parameters to forecast solar power production and the performance ratio of a 2 MWp solar PV project in India [26]. Forecasting of power production from RES is a useful tool for predicting their performance and can enable control and optimization of the loads in a renewable energy system.

Wind energy is another RES that is regularly utilized in off-grid systems. The factors affecting the efficiency of wind turbines are numerous, and they can include ice accumulation, component performance, wind speed, blade length, air density, and rain [27]. For the Nordic cold climate, ice accumulation on wind turbines is a real problem and various attempts at wind turbine ice modeling have been performed [28]. Other challenges besides ice accumulation are high humidity and rain, which were also confirmed by CFD modeling [29]. The simulation showed that on a foggy day, water droplets in the high-humidity air are collected and form a water film on the turbine surface, which results in higher drag and impaired turbine performance. Hence, these factors need to be considered when introducing wind power into an energy system.

Various methodologies can be used to examine the performance of an off-grid energy system. Different approaches for analyzing electricity supply in off-grid systems can include indicator-based analysis, where levelized cost of supply, the weighted score system, and sustainability indicators are important; optimization technologies; multi-criteria decision-making tools and practice-based methods [30]**.** For example, the optimal sizing of various components (PV/wind) with or without energy storage solutions has been performed using different optimization scenarios, both regarding technical and economic parameters [31]. In a power distribution system, the voltage frequencies must be kept within a designed span to avoid damage and blackouts. An imbalance between production and consumption will generally cause problems when implementing more renewable energy, and such problems could be caused by, e.g., a sudden gust at a windmill. Hence, a crucial issue to consider when integrating RES into the electrical power mix is power system oscillations. Several strategies to control and/or dampen these frequency disturbances have been proposed, including maximum power point tracking (MPPT), phase lock loop, power system stabilizers, and artificial intelligence damping controller systems [32]. For example, the MPPT scheme is a commonly used approach in grid connection studies to enhance the power output, and various optimized MPPT strategies have been suggested [33,34]. Power system oscillations are especially noticed when high penetration of RES into the grid and larger off-grid systems occur, and to a lower extent in smaller off-grid systems (e.g., nano- and picogrids), since PV panels and wind turbines are often directly connected to the battery storage.

In an extensive review analyzing 550 articles on PV/wind hybrid systems, it was concluded that most of the research (85.7 %) used simulation tools for their research, while only 5.8 % performed experimental studies. In 8.3 % of the cases, a combination of the two methodologies was presented [35]. The reasons for applying simulation methods were proposed to be both economic and time-related since building up an operational experimental system is both expensive and time-consuming. However, with new methodologies continuously developing, the possibility for experimental research has improved. The Internet of Things (IoT) allows for the monitoring, gathering, and processing of data obtained from diverse types of sensors, machines, and objects in a real-time fashion. For example, to follow up the electricity produced and consumed in real-time in off-grid systems, Raspberry Pi (RPi) and Arduino, both popular and low-cost microcontroller boards, can be used. RPi has previously been utilized in several case studies, e.g., to monitor various parameters from windmills [36] and to gather electrical/environmental data from PV modules [37,38]. Also, in a small off-grid system in Spain, Arduino was utilized to monitor data from a small PV system and a weather station [39]. In addition to this, Arduino and RPi are interesting alternatives for educational institutions since they are both cost-effective and easy to use [40]. Altogether, these studies show the potential of utilizing IoT for monitoring environmental and electrical parameters obtained from RES.

Living labs can be described as a physical or virtual environment where different stakeholders come together to solve real-life problems. The key characteristics of living labs have been thoroughly explored [41]. In this systematic literature review, Hossain and coworkers point out several crucial characteristics important for living labs. These include real-life environments; sustainability; innovation outcomes; stakeholders; business models and networks; methods, tools, and approaches; activities and challenges. The living lab as a concept is indeed large and complex, and several different collaborators, processes and technologies are generally required to push the research forward and/or to develop new products and services. Living labs have also been defined as open-innovation networks, which are differentiated based on four distinct types of actors [42]. Here, universities and educational institutes are suggested to belong to the provider-driven living labs, with an intent to increase knowledge, conduct problem-solving strategies, and promote research. Living labs in higher education can function as an effective tool for cooperation across disciplines but can also address important issues such as sustainability [43].

### 1.2 Contribution and paper organization

To our knowledge, there is a gap in the energy research field regarding off-grid systems in a cold environment using IoT technology. This paper introduces the IoT concept in a nano-sized off-grid location in Finland. An important contribution is that this research is based on real IoT data and that it also presents the problems that can occur in data gathering and transmission processes when studying an energy system with such technologies. Also, the climatic environment in Northern Europe is of great interest since the winters are usually cold. This makes the energy system more vulnerable and creates challenges as there is a seasonal mismatch of solar PV generation and space heating demand. This paper describes and addresses these challenges. Finally, the study has developed a custom-made visualization tool that presents the data collected at the case site. The web-based tool is open to the public and educational institutions, which also makes the Meteoria visitor center an open platform for energy system studies. Since the Meteoria case site is uninhabited for longer periods, there are great possibilities to adjust the energy demand and be flexible in the experimental set-up and investigation. Furthermore, the case site is being continuously developed in an environment that is supported by the local community and several educational institutions in the region, which makes the case site an interesting place for cooperation. Hence, this paper has various goals. Firstly, a technical overview of the off-grid case site and the energy supply, storage, and utilization systems at the Meteoria is provided. Secondly, the software and hardware of the IoT solutions are described and the successful implementation of these technologies is demonstrated. Thirdly, examples of real-time measurements and future possibilities are presented and finally, the current challenges and problems at the Meteoria regarding the off-grid system due to the cold climate surroundings are discussed. The remainder of the paper is organized as follows: Section 1.3 describes the case site. Section 2 presents the development of the energy system and IoT technologies at the visitor center, and the methodology for analysis of various parameters, climatic conditions, and data gathering. Experimental results are described in section 3. Finally, section 4 provides a concluding remark on challenges at the case site and future implications at the Meteoria, and forthcoming work.

### 1.3 Site description

Söderfjärden (63.0°N, 21.6°E) is in the Ostrobothnia region in Finland. Söderfjärden is the result of a meteorite impact that occurred 520 million years ago. It is nowadays a protected Natura 2000 area (2300ha), and it is mostly used for agriculture. The site has a rich birdlife, for example, it is a major resting place for cranes in the autumn [44], and various geological findings have been discovered at the location [45,46]. The site has an advantageous placement since it is situated only about 10 km from the city center of Vaasa. In 2008, the development of the Meteoria visitor center was initiated by the local village association (Sundom Bygdeförening rf) and has over the years been extended with further buildings and equipment. Nowadays, the visitor center gives information about the geological history and tectonic uplift at the location, it displays how agriculture has evolved, and facts about cranes and other birdlife are presented. One of the buildings also has a small observatory run by the regional Astronomical Society Vaasa Andromeda ry. In 2022, the Meteoria visitor center was awarded a sustainable travel status by Visit Finland due to its sustainable energy solutions [47].

**Fig. 1:**
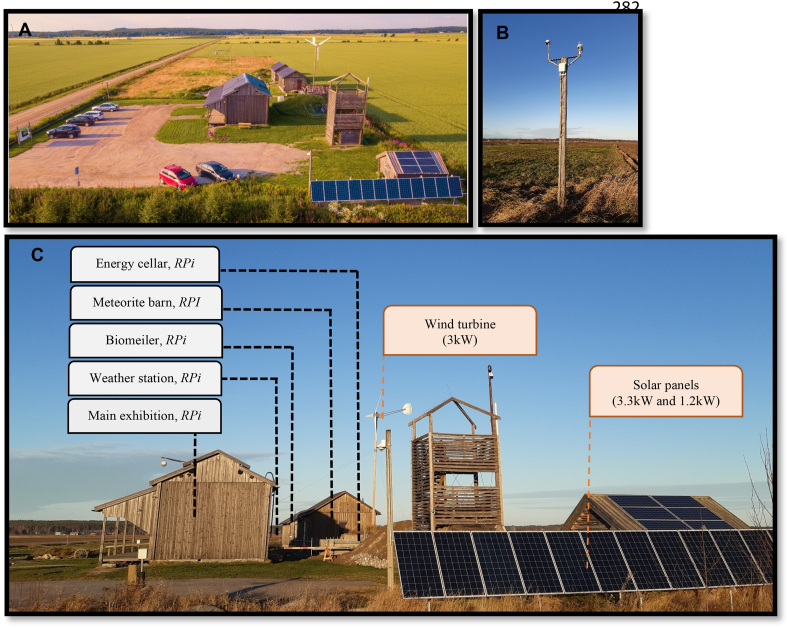
A) An overview of the Meteoria visitor center in Söderfjärden in Finland [48] B) The weather station C) The Meteoria in October 2022. The different buildings, the placements of the five RPis, and the RES are shown.

In the beginning, there was only one building, the *Main exhibition*, a bird-watching tower, and a cellar partly underground next to the main exhibition to hold all energy-related equipment. This place is called the *Energy cellar. S*everal years later, two additional buildings were constructed on the site. One of them is the *Agriculture barn*, which is not heated and it is showing farming tools that were used before the introduction of tractors. The other building is called the *Meteorite barn* and this building is insulated and heated all year around. The meteorite barn holds an exhibition of asteroids and crater-related material. A *Weather station* and a *Biomeiler* have furthermore been introduced at the Meteoria for meteorological data capture and an additional heat source during the cold months. The Meteoria visitor center in Söderfjärden is shown in Fig. 1A and some specific characteristics of the various buildings are displayed in Table 1.

**Table 1:** Specific characteristics of the different buildings at the Meteoria.

	Main exhibition	Meteorite barn	Energy cellar	Agriculture barn	Bird tower
**Size (m**^**2**^**)**	120	42.4	10.5	44.7	10
**Space heating**	No (excess energy for drying)	Yes	No	No	No
**Insulation**	No	Yes	No (underground)	No	No

Until now, the visitor center has been open to the public from the beginning of April until the middle of October, 2 days a week. The open hours are intensified from the middle of June until the middle of August to 7 days a week. Furthermore, groups can book visits at any time of the year. The plan is to be able to arrange visits all year round at the site, and for this to happen there is a need to offer heated spaces and comfort for the visitors (e.g., an indoor toilet). The energy system plays a vital role, and the optimization/development of the energy system processes is of utmost importance. Since the case site may be unmanned for a few weeks, there is a need for self-sustained technologies that can control the system depending on the consumption of electricity and the amount stored, measure the energy production from solar and wind, and automatically start the generator when needed. The IoT methodology installed on this site is imperative for such energy system control and analyses. Over the years, Novia University of Applied Sciences (Novia UAS), Åbo Akademi University (ÅAU), and the University of Vaasa have been involved in the development of the energy system and the setup of the proposed IoT technologies. The planning of the initial off-grid electrical system was presented in 2007 [49], and parts of the energy system have also been touched upon in previous works [[50], [51], [52], [53], [54]]. However, much of the work has been presented in various bachelor's/master's theses and reports, and there is still a lack of detailed description of the IoT solutions and the renewable energy system components developed at the Meteoria. Hence, the goal of this paper is to describe the development and setup of the off-grid energy system and the IoT system at the case site and to make a preliminary analysis of the performance of the energy system for future more thoroughly executed analyses.

## 2 Materials and methods

### 2.1 The development of the energy system 2008–2022

In this study, the development of the off-grid energy system at the Meteoria, consisting of various energy sources and IoT technologies, is outlined. Since the development of the energy system has occurred over several years, the historical background of the components utilized, and the installations performed is depicted to understand the present situation. The buildings and components at the Meteoria in October 2022 are shown in Fig. 1B and C. There are several small buildings, areas, and components of importance at the case site, many of them requiring electricity. In the construction phase of the visitor center in 2008, it was noted that the energy system needed to be an off-grid system since it would have been too expensive to connect the case site to the grid. Furthermore, there was an additional interest for responsible organizations to promote the utilization of RES, to become more sustainable.

At the beginning of 2008, 12 Leader CT200 AGM lead-acid batteries (12V and 200Ah) were implemented to form a 48V and 600Ah energy storage. A 900W Whisper 100 wind turbine, 300W (4 × 75W) of PV panels, and an 11 kW 1-phase 230VAC diesel generator were producing the electricity needed to maintain all the activities for the facilities. The batteries, the wind turbine, and the PV panels have since then been replaced. A Studer XTH 8000-48 charger and inverter were used for generator battery charging and for getting 230VAC from the battery voltage. At that time, there was only the main exhibition at the site and there was no need for space heating. The generator was used as a backup when batteries were discharged and no wind or solar energy was available, and this occurred only during the winter period. In 2013, an additional 1,200W of PV panels (tilted 30°) were added to the system on the roof of a small barn. In September 2016, the wind turbine was changed to a Tuule C200 turbine with a rotor diameter of 5 m, a tower height of 12 m, and a maximum output power of 3,000W. In September 2018, the inverter broke and was replaced with a Victron Quattro 48V 10,000VA inverter, which is still in use today.

In 2018, two new buildings were added to the visitor center. One building shows old agricultural implements (a cold building) and the other is the meteorite barn. The meteorite barn was at that time heated with a Mitsubishi FH25 air heat pump. This heat pump is developed for Nordic climates, meaning it can transfer heat at lower temperatures [55]. The increased need for energy contributed to that 12 polycrystalline 275W PV panels were added to the energy system on a free-standing frame. The frame has a 60° angle from the ground to optimize the energy received from the sun during the winter period. A steeper angle also prevents snow from covering them during the winter. Later in October 2019, the original batteries were renewed to Powerxon 2V AGM lead-acid deep cycle 1,250Ah batteries. Twenty-four of these are connected in series to form the standard 48V voltage. The generator runs nowadays on biodiesel, to be more environmentally friendly. The biomeiler at the Meteoria is a composter that decomposes horse manure and wood chips. Within the composter, water pipes are buried, and if they functioned optimally, they would transfer heat to the meteorite barn [54]. The equipment used in the system, along with some of the parameters, are shown in Table 2.

**Table 2:** Key components in the energy system with some specific characteristics as of August 2022.

Component	Device	Maximum power/energy	Other specifications
PV panels 1	Polycrystalline 275Wx12	3.3 kW	tilted 60°
*Controller 1*	TriStar TS-MPPT-60		Max 60A charge
PV panels 2	Polycrystalline 200Wx6	1.2 kW	tilted 30°
*Controller 2*	Victron MPPT 150/35		max 35A charge
Wind turbine	Tuule C200	3 kW	5 m diameter, 12 m height
*Controller 3*	Finnwind custom made	1.6 kW	
Charger Inverter	Victron Quattro 48/10,000	10 kW	Max 140A charge current
*Control Unit*	Victron CCGX		
Diesel generator	Cummins C11 D5	11 kW	230VAC 1-phase, diesel
Battery storage	Powerxon 2V AGM 1250Ah	60 kWh	About 40 kWh useable
Air source heat pump^a^	Mitsubishi FH25	2.2 kW b	Max 6.3 kW heat, 3.2 kW at −15 °C, used until Sep. 2022
Air source heat pump^a^	Panasonic HZ25XKE	2.0 kW b	Max 7.5 kW heat, 3.6 kW at −25 °C
Biomeiler^a^	50m^3^ compost heater	0–2 kW	Used 2019–2021
Water tank^a^	500l hot water storage		
*Water heater element*		6 kW	Used when batteries are full
*Convector*^a^	Cooper & Hunter	4.6 kW	Connected to both the biomeiler and the water tank
Excess heaters	Space heaters	5.1 kW	Used when batteries are full

a Not the focus of this work.

b Maximum electricity demand.

### 2.2 Description of the IoT solutions

During the first years of operation, one could follow the energy system only locally on-site. No data were logged and if there were problems, they were unnoticed until someone went to the Meteoria. Around 2015, an optical fiber cable for data communication was pulled and connected to the location. Later, in 2019, an IoT development project started, which made it possible to facilitate the build-up of an IoT-based logging system at the Meteoria [56]. The components and IoT devices in the study are shown in Table 3.

**Table 3:** List of IoT devices at the Meteoria as of August 2022.

Component	Device	Connection and communication
**Energy cellar**	Raspberry Pi 3B+	
*Victron system*	Victron CCGX	Modbus TCP
*Inverter and Charger*	Victron Quattro 10kVA	Via Victron CCGX
*PV Panels 1 controller*	Victron MPPT 150/35	Via Victron CCGX
*PV Panels 2 controller*	TriStar TS-MPPT60	Modbus RTU
*Wind turbine energy*	Entes EPR-04S-DIN/RS485	Modbus RTU
*Wind battery charge energy*	AccuEnergy AcuDC243	Modbus RTU
*Generator on/off*	–	Via Victron CCGX
*Generator, energy produced*	Carlo Gavazzi EM111	Modbus RTU
AC, energy consumed	Carlo Gavazzi EM111	Modbus RTU
*Battery monitor*	Victron BMV-700	Via Victron CCGX
**Meteorite Barn**	Raspberry Pi 3B+	
*Air source heat pump control*	Procon MelcoBEMS Mini	Modbus RTU
*Air source heat pump energy*	Carlo Gavazzi EM111	Modbus RTU
*Temperature & RH*	Apar AR252	Modbus RTU
*Convector temperature*	KMTronic LAN DS18B20 - DS1B20 temp. sensors (5pcs)	Modbus TCP 1-wire
***Hot Water Storage***		
*Hot water storage energy*	Carlo Gavazzi EM111	Modbus RTU
*Temperature*	DS18B20 temperature sensor (6pcs)	1-wire
*Controlling*	Marcom MR-RO-1 (7pcs), controls	Modbus RTU
	-Water heater elements 2 kW (3pcs)	
	-Circulation pump	
	-Valves (2pcs)	
	-Radiator cooling fan	
**Main exhibition**	Raspberry Pi 3B+	
*Heat energy*	Carlo Gavazzi EM111	Modbus RTU
*Heater control, 4pcs*	Marcom MR-RO-1	Modbus RTU
*Temperature, 4pcs*	DS18B20 temperature sensors	1-wire
**Weather Station**	Raspberry Pi 3B+	
*Weather station*	Vaisala WXT520	NMEA 0183
*Solar irradiation*	Hukseflux SR30	Modbus RTU
*Ground temperature*	DS18B20 temp. Sensors (10pcs)	1-wire (stopped working)
**Biomeiler**	Raspberry Pi 3B+	
*Biomeiler energy meter*	Kamstrup Multical 602	Modbus RTU
*Circulation pump control*	Marcom MR-RO-1	Modbus RTU
*Compost temperature*	KMTronic LAN DS 18BS20 (3pcs)	Modbus TCP
	- DS18B20 (3 x 5pcs)	1-wire
*Compost temperature*	DS18B20 temp. sensors (7pcs)	1-wire

The Raspberry Pi was selected due to its capability as a general computer using a Linux-based operating system, its small size, and its low power consumption. To promote secure data communication, a reverse VPN tunneling was set up for the main IoT devices on site (RPis). This made the programming and controlling of the system at the Meteoria via the Internet, more easy and secure. During the installation process, it was decided to use several RPis, one per building or location, to get a clearer division of the system. Today, there are five RPis in the Meteoria IoT system, which can be seen in Fig. 1C. In addition, Node-RED was selected as a programming tool for the RPis because it was easy to handle.

**Fig. 2:**
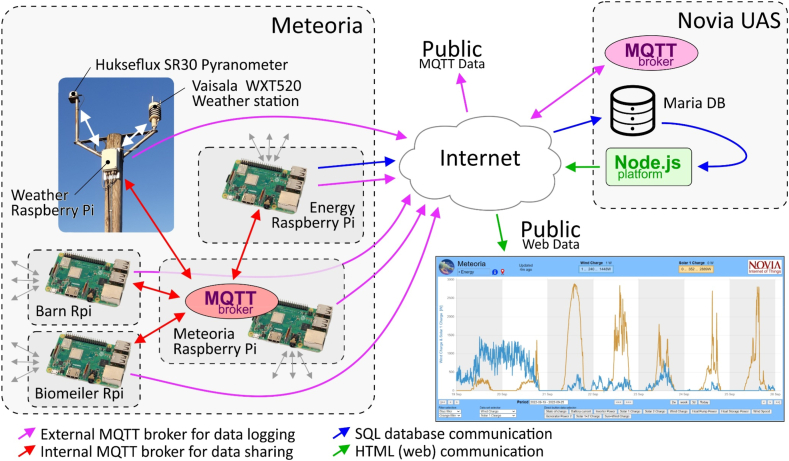
A process scheme of the IoT system at the Meteoria and the data flow in the case study.

Since the energy system was already installed and working at the Meteoria, the IoT system needed to be retrofitted to the available energy system for IoT integration. Several of the devices had a Modbus data communication interface that could be used for reading the data and for controlling the devices. This capability was utilized and USB to RS485 adapters were added to the RPis. This created a small Modbus RTU network for each RPi. Later, when new devices were added, such as energy meters and various sensors, they could utilize the available Modbus RTU networks. Now all the RPis have their own small Modbus network for reading data and controlling the devices within their vicinity. An MQTT (Message Queuing Telemetry Transport) broker in one of the RPis is used for data sharing in-between the devices on site. Another MQTT broker at Novia UAS is used for sharing real-time data with the public. Data are transferred on a minute-based interval to a SQL (Structured Query Language) database at Novia UAS. On the server, a part of the SQL database is publicly available via a visual custom-made data-presenting tool, the Novia IoT platform [57]. Collected real-time data can also be freely subscribed by anyone from the MQTT broker on the same server. The graphical web pages were custom-made in Node-js because there was no available platform during that time that would fulfill the requirements needed. The functionality is basic, data are regularly read from the database, and HTML webpages are created. There is no direct contact from the web user to the database and the data are transferred to the web user via the webpage itself. This makes the loading of the page a bit slow since it includes a down-sampled copy of a part of the database. Once it is downloaded, the response is fast when no further internet communication is needed. The coding of the web pages was done in JavaScript and Chart.js was used for data visualization. The webpages are stored on a server at Novia UAS [57]. Geo-Blocking is unfortunately actively used, therefore there could be limitations to accessing the pages from certain locations in the world. This could be bypassed by using a VPN service. From this visualization tool, various parameters can be selected and followed, e.g., temperature, wind speed, electricity produced from wind, sun, and the generator. Various data sets can be retrieved from October 2019 and forward. Key values are also calculated under the graphical view such as average, peak values, and kilowatt hour (kWh) used and produced during a selected time interval. A schematic of the remote sensor system is depicted in Fig. 2 and an overview of most of the electrical parameters that are measured and saved to the database is found in Table 4. However, in our study, only a few parameters have been analyzed.

**Table 4:** Most of the electrical parameters that are measured in the current IoT system at the Meteoria.

Source	System voltage	Voltage	Current	Power	Energy	Frequency	Complex	Reactive
							power	power
PV-panels 1	120 VDC	no	no	yes	no	–	–	–
PV-panels 1 battery charge	48 VDC	yes	yes	yes	yes	–	–	–
PV-panels 2	120 VDC	yes	yes	no	no	–	–	–
PV-panels 2 battery charge	48 VDC	yes	yes	yes	yes	–	–	–
Wind turbine	250 VAC, 3 phase	no	no	yes	yes	no	no	no
Wind turbine battery charge	48 VDC	yes	yes	yes	yes	–	–	–
Battery	48 VDC	yes	yes	no	no	–	–	–
Local 48 VDC network (Direct from batteries)	48VDC	yes	no	no	no	–	–	–
Diesel generator	230 VAC, 1 phase	yes	yes	yes	yes	no	no	no
Inverter out (Local 230VAC network)	230 VAC, 1 phase	yes	yes	yes	yes	yes	yes	yes
Heat pump	230 VAC, 1 phase	yes	yes	yes	yes	no	yes	yes
Water tank heater	230 VAC, 1 phase	yes	yes	yes	yes	no	no	no
Excess energy (space heaters)	230 VAC, 1 phase	yes	yes	yes	yes	no	no	no

### 2.3 Analysis of the energy utilization at the Meteoria

The electricity produced and consumed at the Meteoria in 2021 was determined in the following way. The total electricity production from the PV panels, the wind turbine, and the generator during 2021 was obtained from the Novia IoT platform [57]. *The heating/cooling of the meteorite barn* is read directly from an energy meter connected to the heat pump, thus, easily extracted from the subsection Barn and parameter Heat pump power (electricity to the air source heat pump in the barn), with the correct period selected. *Excess electricity* is energy dumped to keep the system in balance to prevent the PV panels from stopping to charge the batteries. This installation provides the daily maximum power from the PV panels for statistical purposes. There are two types of excess energy dumping, and each has individual energy meters. One is heating the main exhibition to keep the building dry and healthy, however, during the summer this is not used. The second system is the heating of a 500l water tank. This system is mostly used in the summer period. Hot water could be utilized at night for heating the barn, however, this has not yet been used. From the Novia IoT platform, these values can be found in the subsection Energy and from the parameters Excess Heating Power and Heat Storage Power.

**Fig. 3:**
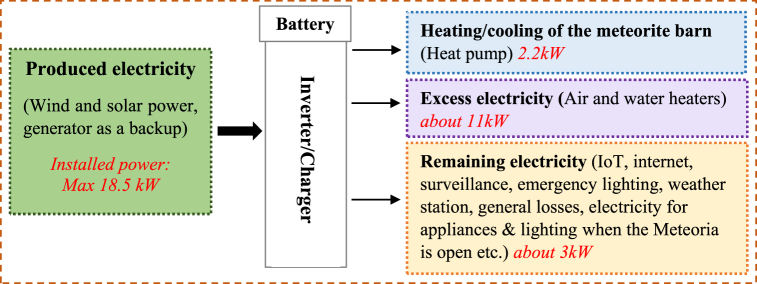
A schematic picture of the produced and utilized electricity at the Meteoria during 2021 expressed as power balance.

*The remaining electricity* is calculated by subtracting excess electricity and heating/cooling of the meteorite barn from all the electricity produced. *Standby consumption* is the base load power used to keep the system working, such as Internet routers, Ethernet switches, RPis, Wi-Fi access points, sensors, emergency exit lights, surveillance cameras, storage for surveillance cameras, and all the energy system devices such as inverters, energy meters, and charge controllers. The standby energy was estimated from the static load when no activity happens at the site and no charging takes place. Several different readings of the standby power during the year were averaged. The average was multiplied by 24 and 365 to get the estimated yearly standby energy. The electricity for the *visitor center activities* refers to when the site is open to the public, which is the purpose of the center. The activities include lighting, video projectors, computers, and a sound system. The electricity utilized is an estimation based on abnormalities during the year in the power consumption when heating and dumping are subtracted. A visitor group is quite easy to recognize from the data, and it can be calculated how many groups have been present and the average power consumption per group. *Conversion losses and others* constitute the final electricity consumption. Fig. 3 displays an overview of how electricity is utilized at the Meteoria.

### 2.4 Analysis of data completeness

The completeness of data was analyzed for several parameters related to the energy system at the Meteoria, listed in Table 5. Data collected for the year 2021 were analyzed, with a data sampling interval of 1 min. The downloaded data from the SQL database were then compared with the data displayed on the Novia IoT platform [57]. It was found that when there is a gap in the recording of data for a parameter, the data points displayed on the visualization platform skip from the point where the recording stopped to the point when the recording resumed. In the downloaded data, the last measured value recorded at the data point when recording stopped is repeated for every consecutive data point until the data point when the recording was resumed is reached. Since no distinguishable value, such as a blank or null value, is given when the recording of data ceases, it is not possible to easily analyze the data completeness by counting the number of blank/null values for a parameter and comparing it to the total amount of data points collected during the year. Consequently, the data completeness was instead analyzed by identifying periods where constant values were recorded for 60 min or longer for three parameters that usually do not stay constant: Irradiance, Wind Speed, and Outside Temp. When these values are constant, it is possible to tell if there is a problem with the recording of data from the weather station or the entire system. A similar check was done for the parameter Inverter Power, and no additional periods where data collection was stopped were found.

**Table 5:** Some parameters used in the analysis of data completeness.

Parameters	Description
Solar Charge 1	PV panels 60°, the output power from the controller, fed into the batteries
Solar Charge 2	PV panels 30°, the output power from the controller, fed into the batteries
Irradiance	The solar irradiance measured by the weather station (horizontal)
Wind Power	The electricity that the wind turbine produces
Wind Charge	The output power from the wind turbine controller, fed into the batteries
Wind Speed	The wind speed measured by the weather station
Outside Temp	The outside temperature measured by the weather station
Generator Power	The output power of the diesel generator
Inverter Power	The total 230AC power out from the inverter
Heat Pump Power	Electricity for heating and cooling the meteorite barn
Heat Storage Power	Electricity for the hot water storage (= excess electricity)
Excess Heating Power	Electricity for convection heaters in the main exhibition (= excess electricity)
Temperature	The inside temperature in the meteorite barn

### 2.5 Analysis of the climatic conditions and the wind and irradiation parameters

To examine the climatic conditions in Vaasa during 2019–2021, the monthly average temperatures were gathered from the Finnish Meteorological Institute (FMI) [58], together with the 30-year average in 1991–2020 [59]. The measured outside and the inside barn temperatures at the case site were extracted from the SQL database using the parameters Outside temperature and Temperature with the right period selected. The monthly temperatures measured by the FMI at the Vaasa airport during 2021 were taken from Ref. [60]. The irradiance data were extracted from the weather station using the SQL database with the year 2021 as a selected period. For analysis of the wind power system, the parameter Wind speed was extracted from the SQL database with the right time selected. The official wind speeds at the Vaasa Airport were extracted from FMI [60].

## 3 Results and discussion

This work describes the development of a nano-size off-grid hybrid energy system (PV/wind/biodiesel) complemented with IoT technologies to facilitate the real-time reading of various parameters, including environmental and electrical variables. The main objective of this paper is to depict the installed components and processes, perform a preliminary examination of the reliability and performance of the IoT solutions, and characterize various specific data-collecting parameters in the energy system.

### 3.1 Justification for the energy system and the IoT technologies at the Meteoria

The decision to promote an off-grid system at the case site was based on several reasons. The most prominent reason was cost-related since it would have been too expensive to build the infrastructure for connecting the site to the grid in the region. Furthermore, the wish to contribute to a more sustainable society as well as the site being situated at a place of great wind and sun possibilities, made the decision easy to turn to RES. In Finland, there is now an enormous capacity for wind development projects. At the end of 2022, the completion of 1,393 installed wind turbines had been executed, with a total capacity of 5,677 MW [61]. Of these, Ostrobothnia retained 16 % of the cumulative wind power capacity. Also, the majority of the planned wind-power projects are set to reside in western Finland, with Northern Ostrobothnia and Ostrobothnia as the two top leading regions [62]. This shows that the Ostrobothnia region is a favorable location for commercial wind power plants. Installed solar power production capacity is also growing, however, from a modest level. Grid-connected solar power capacity in Finland grew by about 34 % during 2021, to 395 MW. Besides this, it has been estimated that about 22 MW of off-grid PV panels are installed in around 55,000 houses and summer cottages in Finland [63]. The sunshine hours in Finland vary with the latitude. A report by the FMI has stated the total yearly sunshine hours for various locations in Finland using the average monthly means measured during the period 1991–2020. The total yearly sunshine hours were 1,253h for Utsjoki (69.3°N, 27.0°E), 1,718h for Seinäjoki (62.93°N, 22.5°E), and 1,890h for Helsinki (60.23°N, 25.0°E) [64]. In the same study, the yearly sunshine hours were not measured at the Vaasa observation site. However, since Vaasa resides at 63.1°N, 21.6°E, the yearly sunshine hours for Vaasa can be approximated to about 1,600–1,700h. Available solar radiation decreases radically during the winter months in Finland, but there is also a significant difference between distinct locations in the country. For example, in 2012, the annual solar irradiation was 1095 kWh/m^2^ in Helsinki, 892 kWh/m^2^ in Jyväskylä, and 858 kWh/m^2^ in Sodankylä [65]. In conclusion, considering all these facts, the starting position to utilize both wind- and solar power as RES at the Meteoria is considered satisfactory.

**Fig. 4:**
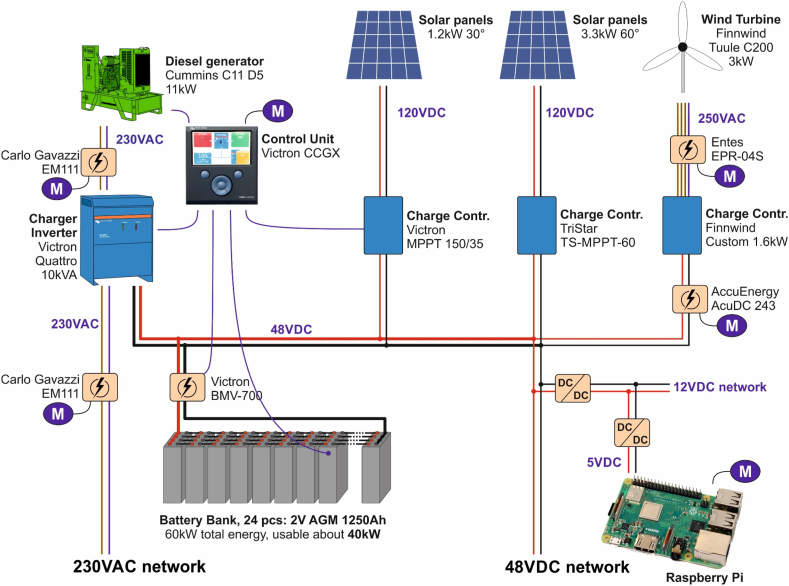
A schematic picture of the system in the energy cellar in 2021, showing the charge controllers for the RES, the diesel generator, the batteries, the inverter, and the IoT system. The M in the picture indicates the connection to the Modbus RTU and TCP data networks used by the RPi to read and control the system.

To understand an energy system, several crucial parameters, such as the various utilized energy sources and their interaction, need to be evaluated. This can be performed by modeling and simulation methods [35] but also by examining gathered energy data obtained from sensors and IoT technologies. Obtaining real-time data could give imperative information on unexpected events, such as component malfunction or sudden changes in weather conditions. In this study, all devices in the system have a digital data communication interface and are read digitally to the RPis. Additional energy meters and IoT sensors have been installed to get complete coverage of the system. The energy data are stored on a server at Novia UAS and visualized on a platform openly available to the public. Worth noting is that the energy system, containing RES, a generator, and battery storage was already in place when the IoT technology was retrofitted to the energy system. The key components of the energy system are in the energy cellar (Fig. 4). The cellar is underground and has an approximate temperature between 0 and 15 °C during the entire year. Nevertheless, during chilly winter days (ambient temperature below −20 °C), the temperature in the cellar can decrease below zero degrees. However, this happens very rarely, and the equipment in the cellar does not presuppose any space heating. In the cellar reside the batteries, the diesel generator, the controllers for the PV panels and for the wind turbine. The controllers convert the voltage to the 48V battery voltage as well as protect the batteries from overcharging. If the battery discharges until a critical level, the RPi in the energy cellar will automatically start the generator until the batteries are almost fully charged. To get a clearer picture of how the system is behaving remotely, additional energy meters and sensors have been added to the system. Via reverse VPN tunneling in all the RPIs, the system can be fully read, controlled, or updated remotely, for example, to manually run the generator and the heating system or to reprogram the behavior of the system. The other locations for the RPis at the Meteoria are the weather station, the meteorite barn, the main exhibition, and the biomeiler (Fig. 1B and C).

### 3.2 Analysis of electricity production and consumption at the Meteoria

To maintain the activities at the case site, electricity is required for various processes, which are also depicted in Fig. 3. The main consumer of electricity is heating/cooling the meteorite barn, which is open to the public for seminars and events from April–October. The heating (or cooling) during 2021 was performed by an air source heat pump, suitable for Nordic climates, meaning it can operate at lower temperatures (until about −20 °C). During winter, the temperature maintained in the barn has been flexible depending on the available energy, though it has been desirable to keep the inside temperature over the freezing point. Another prioritized area of energy consumption is the standby electricity system, such as the IoT system, the fiber internet connection and the ethernet network, Wi-Fi, surveillance cameras, controllers, and sensors. These enable the possibility to monitor the electricity generation/utilization as well as directly control the generator (backup source). Electricity for appliances & lighting is needed when the visitor center is open for events (visitor center activities). These activities include lighting, computers, and projectors (for showing videos and making other presentations). During some periods (when there is a surplus of solar and wind power), excess energy is produced that cannot be stored in the batteries. To get the maximum available power from the wind turbine and the PV panels, the system needs to be in balance to avoid the batteries becoming full and the controllers stopping to charge the batteries. This is done to obtain the total energy produced by the wind turbine and the PV panels. For this reason, the convection heaters in the main exhibition are automatically activated by the IoT system. The convection heaters warm up the building slightly and lower the relative humidity to keep the building healthier during spring and autumn. During the warm summer months, the heating of the main exhibition is not desired. Therefore, there is another system to transfer the excess electricity. A heat storage water tank next to the meteorite barn can then be used for dumping the excess electricity. Currently, these processes are not optimized, however, they give future possibilities for utilizing the excess electricity produced during favorable climatic conditions.

To examine in more detail the electricity production and consumption at the Meteoria, data from the available sensors/controllers were analyzed, which is depicted in Table 6. During 2021, about 7,400 kWh of electricity was generated, with solar power as the largest contributor (62 %), and wind power as the second largest (30 %). These data can directly be extracted from the PV panel controllers and an external charging energy meter for the wind turbine. However, there was a minor gap in the production from the PV panels because the smaller PV panels had a fuse that was blown (also discussed in Table 7). The generator produced a minor amount of the total electricity (8 %). Currently, there are not enough energy meters installed at the Meteoria to be able to analyze the consumed electricity in detail by consumption category. The heating/cooling of the meteorite barn (by the heat pump) is measured with an energy meter, and the result showed that these processes consumed about 26 % of the electricity produced during 2021. The excess electricity produced is furthermore measured with energy meters, and the result indicated that 30 % of electricity production is used for this. This large amount of excess electricity is an interesting detail, and this will be an area of optimization in the future. Although the energy system at the Meteoria is a system with defined system boundaries, the several components and devices within the system make it quite complex. Thus, measuring all relevant electricity data would require further sensors and devices installed compared to what today's system contains. Consequently, in this study, some data are estimated, such as the remaining electricity consumption. One interesting note in these results is that only about 6 % of the total electricity consumption was utilized when the Meteoria was open to the public, which is the main purpose of the Meteoria visitor center activities.

**Table 6:** The amount of electricity utilized at the Meteoria during 2021.

Electricity	Produced		Consumed	
	kWh	%	kWh	%
Solar	4,606	62		
Wind	2,231	30		
Diesel	558	8		
**Total**	**7,395**	**100**		
Heating/cooling of the meteorite barn			1,955	26
Excess electricity			2,251	30
Remaining electricity			3,189	43
* *Standby consumption*			*2,155*	*29*
* *Visitor center activities*			*455*	*6*
* *Conversion losses and other*			*579*	*8*
**Total**			**7,395**	**100**

*Estimated data.

**Table 7:** Identified problems in the data transmission at the Meteoria during 2021.

Type of problem	Components/parameters involved	Frequency and/or duration
**1** Measurement interval ≥2 min	All sensors simultaneously	About 70 occurrences
**2** Two values are recorded per minute	All sensors simultaneously	Occurs on one occasion for 1 h.
**3** Missing data: constant values are recorded for parameters that tend to fluctuate	Weather station (Wind Speed, Outside Temp, Irradiance), *Solar Charge 2*, Wind Speed	The weather station: 15 days, *Solar Charge 2: 13 days* (each one occasion) Wind Speed: 6 days
**4** Missing data: constant values are recorded for every parameter	All sensors simultaneously	Occurred on four separate occasions for a total of about 22 h.
**5** Erroneous values recorded	Wind Power	Wind Power spiked to 9999.9 on three occasions for a total of 5 min
**6** Contradictory data	Wind Speed, Wind Power	About 9 days

**Fig. 5:**
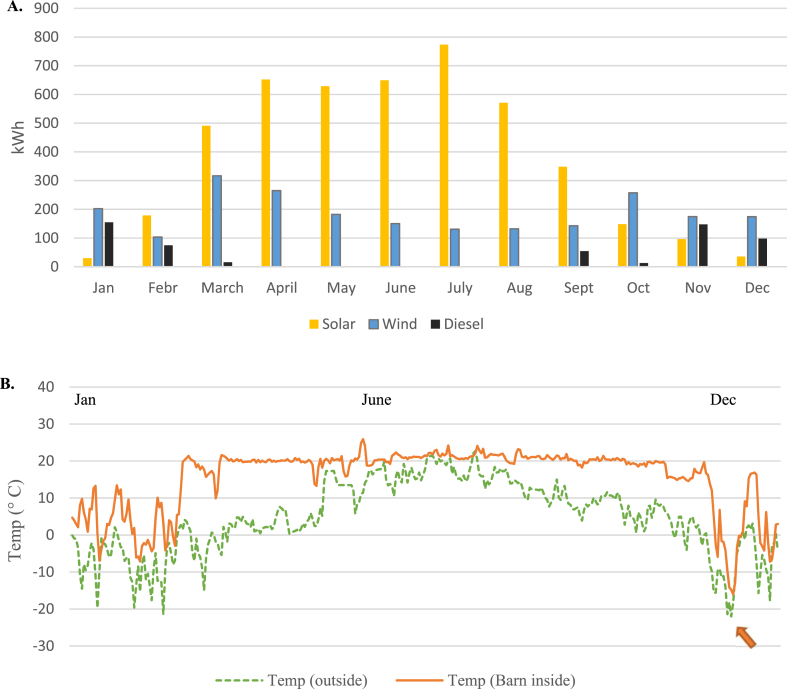
A) The average monthly production of various energy sources at the Meteoria during 2021 B) The average daily outside and inside barn temperatures in 2021 at the Meteoria as measured by the installed sensors. The arrow highlights a challenging situation.

To demonstrate the availability of the different energy sources at the Meteoria during 2021, the average monthly energy production is shown in Fig. 5A. The results show that solar energy is mostly available from March to August, wind energy is more evenly distributed throughout the year, and the diesel generator runs mostly during the winter months (Nov–Feb). In Finland, the warmest months are June to August, which also mostly coincides with the opening hours at the Meteoria. This is an advantage, however, the results in Table 6 indicate that only 6 % of the total electricity demand is needed for the Meteoria visitor center activities. About 26 % of the total electricity demand is directed to heating/cooling the meteorite barn. Especially during the winter months (Nov–Feb), there is a need to try to keep the inside temperature above zero degrees. This is why the diesel generator is sometimes utilized during the colder months. The results in Fig. 5B depict how the inside temperature is affected by the outside temperature. For example, at the turn of the months of Nov–Dec, there was a sudden change in the outside temperature to below −15 °C, with the consequence of the inside temperature dropping below zero degrees for a couple of days, despite the diesel generator being in use. Increased utilization of diesel generator power could be one solution to combat the unwanted inside temperature drops. However, the high costs of such fuel consumption as well as the desire for managing with 100 % sustainable energy solutions, indicate that there might be a need for long-duration energy storage and more RE sources at the Meteoria. Furthermore, the possibility to lower the energy demand during challenging periods is presently an advantage of this demo site. Nevertheless, the level of energy supply required to maintain the desired temperatures could be clarified in future studies. In conclusion, the analysis performed here suggests that to be able to extract more detailed data, further energy meters and sensors need to be installed. However, it is important to decide whether the benefit of having a more detailed system is larger than the possible challenges that might evolve with the increased complexity of the system.

### 3.3 Analysis of data monitoring and transmission

To illustrate the performance of various devices and the reliability of data transfer over a longer period, an analysis of the amount of missing data or data transmission problems during the year 2021 was performed. Not all available parameters were analyzed. Instead, only those mentioned in Table 5 were selected to identify potential problems in the data communication and transmission. The parameters for which problems were identified were the sensors in the weather station, the sensors at the controllers for the wind turbine, and the PV panels. Furthermore, there are gaps in the data involving all parameters. The results revealed temporary sensor failures and several interruptions in the data communication and transmission, with six main problems identified **(**Table 7).

For one year, 523,592 measured data points were identified. There are 525,600 min in a year, so approximately 2,000 of the annual data points were missing, and thus, 99.6 % of the expected amount of data points were collected for the year. This is due to the first two types of problems identified, which are affecting the number of data points collected. Problem 1 was that the sampling interval was sometimes longer than 1 min, leading to fewer data points being recorded. The gap in the sampling interval varied from 2 min to hours but was usually between 2 and 10 min. This happened on around 70 occasions in the analyzed data from the year 2021. This type of problem is presumably a result of momentary internet disruptions between the devices at the Meteoria and the database at Novia UAS. The second problem noticed to affect the number of data points collected (problem 2) was that two measurements were performed in a few seconds within the same minute, adding superfluous data points. However, this occurred only on one occasion for 1 h, and the most probable cause is suggested to be due to reprogramming and debugging taking place during this time.

The third problem observed (problem 3) was that data were missing from some of the sensors. For example, a period in May 2021 there was no recording from the sensors of the weather station for 15 days, and in June there was a gap in the measurements from one set of the PV panels at the site (SolarCharge2) for 13 days. The DC power supply converter from 48V to 12V at the weather station broke, and it took some time to get the spare part and have it replaced. This resulted in 15 days of missing data from the weather station. For the missing data points for the PV panels in June 2021, a further examination showed that a fuse was blown during that period, which consequently contributed to the PV panels not contributing at all to the electricity production. However, the data communication was still functioning as intended. Furthermore, in February 2021, there were several gaps (about 6 days) in the wind speed data, which are discussed further in chapter 3.5.

The fourth problem identified (problem 4) was when all the data became static. Based on the analysis done in this study, this occurred four times with a total duration of about 22 h. Normally, the generator starts when needed, but for distinct reasons, the generator did not start four times during that year. At these times, the battery capacity soon became critically low. To protect the batteries, the inverter shut down and there was a blackout in the 230V network. The IoT system runs directly on the 48V system and is not affected by this. During the years of extension and changes of the system at the Meteoria, some Ethernet switches used 230VAC. During these blackouts, part of the Ethernet network was turned off and major communication failures resulted in these periods of missing data. This problem was corrected during the year 2021, and today, the complete IoT, Ethernet, and Internet connection are run on 48V directly from the battery.

Erroneous values (problem 5) are undoubtedly difficult to recognize since this means that you usually need a comparison with a reference, which you are sure is correct. However, in the data analyzed, there were examples of erroneous values appearing. For the parameter Wind power, the values suddenly spiked to 9,999.9W from zero on one occasion. This value was substantially different from the values recorded before and after these occurrences. The value 9,999.9 is the maximum value defined in the database. After a thorough examination of this error, it was concluded to be due to software updates in one of the RPis. The IoT data system is an asynchronous system and data are not read from all the devices in the system at the same time. When the software in one of the RPis is updated, it can be that a database insert is performed before the data from some of the other RPis sub-devices become available to the one doing the database insert. Measures have now been taken to avoid this problem. However, there can also be other unknown bugs or complications in the system that could generate these kinds of problems. An additional problem (problem 6) was identified on several occasions regarding the wind speed and the electricity production from the wind turbine. Here, it was shown that although the wind speed parameter showed a wind speed over 5 m/s (which should be enough to start electricity generation since data from the site show that this normally happens at 2.5–3 m/s), the data from the wind turbine controller showed zero, indicating no electricity production. To date, the reason for this occurrence is unknown and the phenomenon needs to be investigated further.

In an off-grid community in Brochet, Canada, a data completeness analysis was performed on several meteorological variables, such as wind speed, direction, and temperature for one year [66]. The hourly data and the number of gaps (denoted by blank cells) were analyzed by comparison to the total hours in a year, and it was concluded that the missing values ranged from a single value to a maximum of 72h. In the case study here, with a data-measuring interval of 1 min and close to 100 different devices dependent on each other for the system to work, the data received for one year are enormous. It is therefore presumable that there are gaps in the data transmission and that it will occur for distinct reasons. Considering that the IoT system has been retrofitted to the energy system, and both the energy system, as well as the IoT technologies are continuously updated, there are relatively few problems occurring. Here, alteration in the measuring interval and the breaking of components could be observed. However, it should be noted that the recording of data for these parameters may have stopped for shorter than 60 min and that every individual parameter may have stopped recording data for an unknown period. Furthermore, it is not possible to tell for many parameters as some of them tend to remain constant most of the time. However, it can be concluded that the data are reasonably complete and that it will make a reliable data source for further investigation.

Nonetheless, there might be other limitations in the current IoT system. Currently, the data are collected once per minute, which in this study is considered enough. However, if a much faster sampling rate is desired, there might be problems in transferring the data points individually via the MQTT broker and in the Node-RED environment. Also, the Modbus RTU that is used to read most of the devices is quite slow and sampling rates closer to a few seconds could become challenging when several devices use the same bus. Data at the Meteoria are not sampled synchronously from all the devices at the same time, instead, the devices are sampled randomly within the 1-min sampling rate. This needs to be considered when analyzing the data more in-depth than in this study. When several devices share the same Modbus RTU bus, synchronous sampling is not possible since only one request at a time is possible. In addition, in the current IoT system, there is no automatic feedback system to the administrator, e.g., when components break or experience problems. Since the off-grid system is unmanned for extended periods, such an upgrade would be beneficial and easy to implement in an IoT system.

### 3.4 The climatic conditions at the case site

**Fig. 6:**
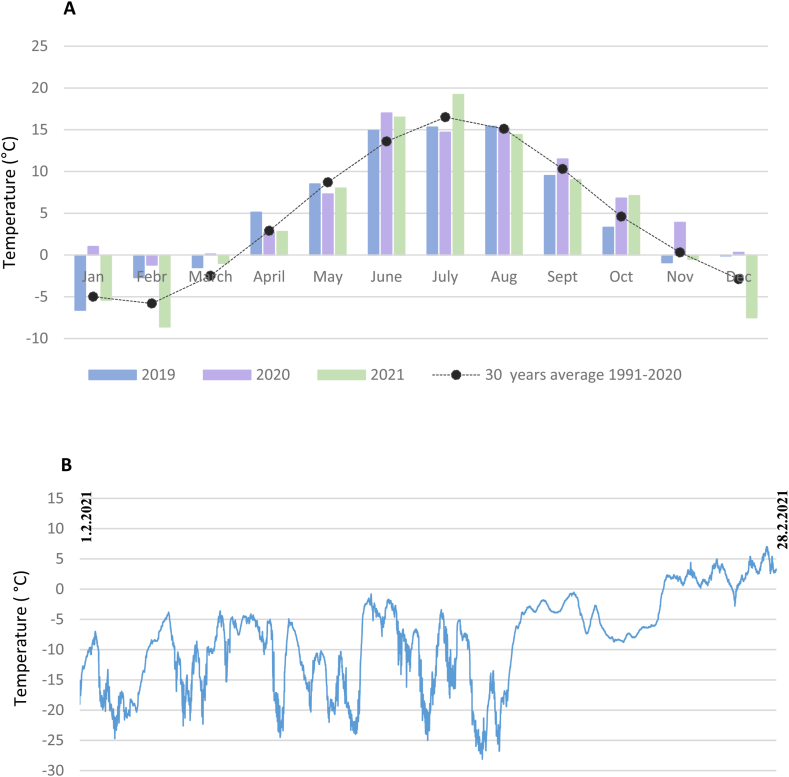
A) The average monthly temperatures (°C) and the 30 years average 1991–2020 in Vaasa, Finland, measured by the FMI [58,59] B) The temperature in February 2021 at the Meteoria as measured by the weather station.

One crucial parameter in an off grid-system is the climate surrounding the energy system. For example, the efficiency of PV panels is affected by the temperature [21], and the wind turbines by humidity and wind speed possibilities [27]. The city of Vaasa in Finland resides in Northern Europe at latitude 63.10°N, which means that Finland belongs to the continental subarctic or boreal climates according to the Köppen climate classification. In practice, this means that the summer months are generally warm to cool, and the winter months are cold. However, the temperatures might vary, which is also demonstrated in Fig. 6, where the temperatures in Vaasa are displayed. In Fig. 6A, the average temperatures per month in Vaasa (2019–2021), as measured by FMI [58,59], are shown. In the Ostrobothnia region where Vaasa resides, these temperature values are normal with the warmest months in June to August, and December to February as the coldest months. Furthermore, between distinct years, there can be a substantial difference in temperatures, especially during the cold months, which is also observed, for example, during January, February, and December. Fig. 6B demonstrates the temperature in February 2021, measured by the weather station (measuring at 1-min intervals) at the Meteoria. The results show the continuous measuring of the outside temperature at the case site, with temperatures sometimes touching −25 °C but with an average temperature of −8.4 °C.

To verify that the temperature sensor installed at the weather station at the Meteoria is functioning as intended, an analysis of the temperature sensor installed in comparison to the official temperatures at the Vaasa airport measured by the FMI was performed (Table 8). The monthly temperature averages match each other quite well. However, in May, there was a larger gap in the transmission from the weather station (see Table 7, problem 3), which is also demonstrated in these results. The other slight differences between the two measuring devices can probably be explained by the distinct locations (about 10 km) of the measurements and small possible breaks in the data transmission (Table 7).

**Table 8:** The monthly average, minimum, and maximum temperatures (°C) in 2021 were measured by the weather station at the Meteoria, and the average monthly temperatures as measured by FMI [60].

		Meteoria		Vaasa Airport (FMI)
2021	Min	Max	Mean	Mean
January	*−23.1*	*2.9*	**−5.3**	**−5.4**
February	*−28.1*	*7.0*	**−8.4**	**−8.6**
March	*−22.8*	*9.6*	**−1.0**	**−1.0**
April	*−3.5*	*16.2*	**2.8**	**2.8**
May	*−3.4*	*19.2*	**10.4**^a^	**8.0**
June	*4.6*	*25.0*	**16.0**	**16.5**
July	*5.9*	*30.2*	**18.5**	**19.2**
August	*1.2*	*24.2*	**14.3**	**14.4**
September	*−1.5*	*18.1*	**9.0**	**9.0**
October	*−3.5*	*14.7*	**7.2**	**7.1**
November	*−21.1*	*9.6*	**0.0**	**−0.5**
December	*−27.0*	*4.4*	**−7.4**	**−7.5**

a *Sensor out of order 12-27 May 2021*.

In summary, these outcomes show that the temperature might differ substantially between years, months, and within one month. This benchmark might influence the energy system at the case site, as the amount of energy required varies with the temperature, with more energy needed during colder periods. Furthermore, we conclude that the sensor at the weather station gives stable and reliable values, although technical issues might occasionally appear.

**Fig. 7:**
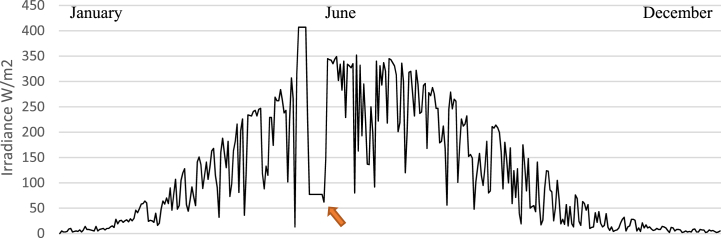
The irradiance at the Meteoria 2021 was measured with the Hukseflux SR30 pyranometer (horizontal). The values are shown as daily averages. The arrow shows a disruption in the data communication from the weather station between 12 and 27 May 2021.

In addition to the large temperature variations in Finland, Northern Europe experiences a considerable variation in solar irradiance for one year [65]. To demonstrate this and to examine the measurements from the irradiance sensor (horizontal measurements), the Hukseflux pyranometer, the daily average irradiance (W/m^2^) in 2021 is depicted in Fig. 7. The results show that the winter months (December–February) have low incoming solar energy, but the summer months (May–August) have higher irradiance. Although PV panels are usually suggested to be more effective when it is not very hot [20], locations in Northern Europe will experience other challenges, such as low irradiance during the winter months. These months are naturally the coldest months, which will further put constraints on the energy system, especially if energy is needed for heating buildings. This demonstrates the importance of appropriate long-term energy storage solutions for balancing energy production/consumption during challenging climatic times.

### 3.5 Preliminary characterization of wind parameters

An initial characterization of the wind prerequisites was made based on data obtained from sensors installed at the Meteoria. From the weather station, wind speed data from 2021 were extracted and the results are depicted in Table 9.

**Table 9:** The wind speed (m/s) as measured by the weather station at the Meteoria and by the FMI at Vaasa airport [60].

Year	Meteoria Wind speed (m/s)			Vaasa Airport Wind speed (m/s)
2021	Min	Max	Mean	Mean
January	0.0	19.4	**4.1**	**3.2**
February	0.1	29.4	**7.0**^a^	**2.7**
March	0.1	17.9	**4.6**	**4.2**
April	0.0	33.4	**4.3**	**4.2**
May	0.1	12.8	**3.5**	**3.6**
June	0.1	18.4	**3.1**	**3.5**
July	0.0	11.3	**2.6**	**3.3**
August	0.1	22.1	**2.9**	**3.3**
September	0.0	21.6	**3.0**	**3.0**
October	0.0	23.9	**4.3**	**4.4**
November	0.0	30.1	**3.3**	**3.0**
December	0.1	23.3	**4.6**	**3.1**

a *Sensor problem*.

**Fig. 8:**
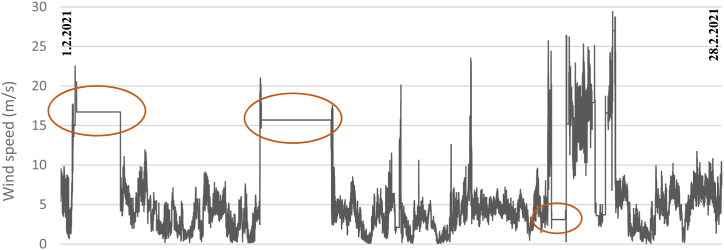
The wind speed (m/s) at the Meteoria in February 2021 was measured by the Vaisala weather station. The orange circles highlight periods with identified problems.

In general, wind speed plays a large role in wind power generation [27] and the parameter is usually very location-dependent. To examine this parameter, the wind speed (m/s) at the Meteoria as a monthly average was compared with official statistics by FMI from Vaasa airport [60]. The results show that although the wind speed is measured at distinct locations (around 10 km apart), the monthly measured average wind speeds follow quite well each other. However, some months display larger variations in wind speed. Especially in February 2021, the wind speed showed a relatively high average value (7.0 m/s) at the Meteoria. Fig. 8 shows the wind speed in more detail, where there are several periods where the wind speed values become static, which was also observed in the analysis shown in Table 7. The parameters of wind speed and wind direction at the Vaisala weather station are measured by a sensor operated with ultrasound, while the temperature, humidity, and pressure parameters are measured by distinct sensors [67]. Since both the wind parameters show similar behavior in February, but not the other parameters (data not shown, except the temperature in Fig. 6B), the most probable reason for this problem is ice accumulation/dust/clogs on the wind sensor or transducer, which is blocking the ultrasound.

Also, worth noting is that the weather station is at a height of 3.5 m, while the wind turbine is at a height of 12 m, which means that the wind speed at the wind turbine and the weather station might slightly differ. One solution to optimize the measurements of the wind speed to better mimic the real conditions at the wind production site is to install a wind speed sensor closer to the wind turbine and at the same height.

## 4 Conclusions and future work

This study presents the development of an off-grid system at the Meteoria and the successful retrofitting of the energy system with IoT technologies. Since the Meteoria is run by non-profitable organizations, the development of the energy system at the Meteoria was not in the beginning based on the most optimal solutions but instead on available and cost-efficient components. However, over the years, components have been changed, removed, or added. Nowadays, there are several IoT solutions available for research, industrial applications, and smart environments. In this study, several programming languages and environments were examined, such as C/C++, Python, and Node-RED. However, during the working process, it became clear that in Node-RED, the IoT devices could be set up within minutes, and Node-RED was therefore selected. Node-RED has previously been proposed in several other studies and is suggested to work well with RPi [68] and with both the Modbus and MQTT protocols [69]. Furthermore, in the switchgear industry, an energy monitoring system based on RPis and Node.js has been used [70], demonstrating their applicability in the industrial sector.

The importance of having a real-time measuring solution at the Meteoria site, providing accurate data, is vast, and such solutions may provide many advantages. Firstly, although simulation methods are in many cases excellent alternatives [35], real-time experimental data will give additional value, since they will help identify abnormal behaviors or unexpected events when they occur. For example, if there are problems concerning the infrastructure, that is the RES, the IoT sensors, or the related equipment, they will be quickly noticed, and the problems can be solved in a proper period. In this study, an attempt to look at potential problems that occur in the data transmission from the off-grid system for one year was performed. Component failures, changed measuring intervals, and static measuring values were identified problems. Several of these occurrences were easily detected in real-time, while others required further investigation. A second advantage was that the data received could later be analyzed, and optimization of the energy system could be performed to be more efficient and smoothly working. Thirdly, since this case site is cooperating well with regional higher education institutions, the possibility of creating the Meteoria into a test bed or a living lab environment is possible. Since a living lab usually contains several stakeholders, one crucial element is to enable an uncomplicated visualization of results for both the partners and the public. Grafana is one example of an open-source platform that has previously been used in a study on applying IoT to investigate a solar plant monitoring system [71]. In this paper, another custom-made, easy-to-use visualization tool is presented, and this is openly available for the public and educational institutions. The Meteoria visitor center is an off-grid site aiming at promoting 100 % renewable energy utilization and thus raising awareness of sustainable energy solutions among various disciplines. The results from this study show an off-grid energy system that has successfully been retrofitted with IoT technologies. The graphical visualization tool makes it easy for all interested, including students, partners, and citizens, to follow various electrical and environmental parameters and gain insight into a complex energy system. Until now, several regional educational institutions have been involved in the build-up and development of the Meteoria, and both personnel and students have had the opportunity to be engaged in the energy research field. In addition, several foundations and organizations have supported the activities at the Meteoria, which has made the visitor center an important regional location. This case site has no intention of becoming a top-of-the-line research facility but instead a learning environment for promoting sustainability together with the public and educational institutions in the region. This platform will increase knowledge of how energy is produced and consumed in a cold climate, as well as how to find the most optimal energy solutions.

The results of this study indicate several challenges regarding the current energy system at the Meteoria visitor center. The cold winters (Nov–Feb) are challenging since the amount of energy needed to heat the building is higher. One way to address this issue is to install additional energy sources to increase the available energy. Another way would be to install long-term energy storage to be able to save energy from periods with excess energy production to periods with less energy availability. One problem in Northern Europe is the low irradiance occurring during the winter months (Nov–Feb), which is also shown in Fig. 7. This means that there is a shortage of solar energy during the cold winter months, decreasing the energy flow from solar panels. Using solar panels with different angles could be one approach to analyze the possibilities to improve the utilization rate of solar radiation. The ice/snow coverage of the wind turbine and solar panels might provide an additional challenge during the winter months at the Meteoria. However, in this study, no specific sensors detecting snow/ice parameters have been installed but this could be a future study of research. Also, the energy storage capacity in the batteries is reduced at low temperatures, which can influence the results.

In general, challenges with off-grid electrification have been discussed [8]. The initial investments are generally high, and the supply and demand of electricity are often not matching, especially when using intermittent RES such as wind and solar power. In Northern Europe, where the winter months are cold, there is normally a need for both short-energy storage and seasonal energy storage. A study on an off-grid system (a residential house with a ground source heat pump-based heating system) in Finland, based on solar energy and battery- and hydrogen energy storage, has been performed [72]. The proposed system was simulated based on real PV power data, and the result suggested that both modes of energy storage were needed to withstand the long and cold winters. Currently, the Meteoria is not open during the winter months, which eases the requirement of heating the space. However, the aim is to be open during the winter, which makes the optimization of distinct types of energy sources and energy storage more obvious. For example, by looking at the electricity data from 2021 at the Meteoria, it was shown that there is a surplus of electricity production of about 30 %, and this is currently not taken advantage of. Hence, there is room for optimization of the energy system. As this study established that data are being collected reliably by the IoT system at the Meteoria, further improvements to the system could be made by integrating data-based AI techniques into the energy system to take advantage of the collected data. Data-based machine learning models could be developed to forecast the power production from the RES, which will enable better optimization and scheduling of the energy use at the Meteoria. The site is continuously being developed, and during 2022–2023, the meteorite barn will be complemented with an additional exhibition space, a small kitchen, and a dry toilet. The original heat pump has been replaced with two new ones, and a biodiesel cottage heater has been added. This is to avoid running the generator to produce electricity in the winter period for the air source heat pump when it has a low electric energy intake to heat efficiency. At the same time, the energy storage will be complemented with lithium-ion batteries and vertically mounted PV panels, and a new inverter and charger will be installed. These changes will create new optimal energy solutions, give further possibilities for the case site to function as a learning environment and a platform for research, and give excellent opportunities to investigate the off-grid system at the Meteoria. The off-grid energy system at the Meteoria is nano-sized, which means it is about the size of a private household. Also, the fact that the case site is in northern Europe, with specific climatic parameters, such as irradiance patterns and temperature variations, will be of interest to society but also to the private energy consumer. In today's society, there is an increased interest in installing and utilizing renewable energy sources. For the private energy consumer, it might be helpful to be able to follow how various energy sources and energy storage modes might interact with each other. In addition, the results of this study suggest challenges that might occur when using IoT measuring technologies in energy system measurements.

## Funding

For the development of the energy system and IoT solutions at the Meteoria, funding from several sources has been received. These include Merinova, Aktiastiftelsen i Solf-Sundom, the ELMA development project, the LEADER development project, Mervento Power Technology, Teknologiateollisuus ry Länsisuomi, Otto Malms Donationsfond, the ERDF project IoT in energy systems, Högskolestiftelsen i Österbotten, Svenska kulturfonden, Aktiastiftelsen i Vasa, Sundom bygdeförening, and the educational institutions Novia UAS, ÅAU, and 10.13039/501100024027University of Vaasa. For publishing open access, a grant was received from the 10.13039/100016205APC pool, funded by Gösta Branders research fund, Åbo Akademi Research Foundation. The financial support is gratefully acknowledged.

## Data availability statement

Data will be made available on request.

## CRediT authorship contribution statement

**Jessica Tuuf:** Conceptualization, Formal analysis, Investigation, Methodology, Supervision, Validation, Visualization, Writing – original draft, Writing – review & editing. **Hans Lindén:** Conceptualization, Data curation, Formal analysis, Funding acquisition, Investigation, Methodology, Project administration, Resources, Software, Supervision, Validation, Visualization, Writing – original draft, Writing – review & editing. **Sami Lieskoski:** Conceptualization, Formal analysis, Investigation, Methodology, Validation, Visualization, Writing – original draft, Writing – review & editing. **Margareta Björklund-Sänkiaho:** Conceptualization, Formal analysis, Funding acquisition, Investigation, Methodology, Project administration, Resources, Supervision, Validation, Visualization, Writing – original draft, Writing – review & editing.

## Declaration of competing interest

The authors declare that they have no known competing financial interests or personal relationships that could have appeared to influence the work reported in this paper.
